# Efficacy and safety of direct oral anticoagulants versus warfarin in patients with a left ventricular thrombus: an updated systematic review and meta-analysis of randomised controlled trials

**DOI:** 10.1136/openhrt-2025-003542

**Published:** 2025-11-19

**Authors:** Thomaz Alexandre Costa, Gabriel Cavalcante Lima Chagas, Luma Maria Tavares de Sousa, Bruno Lins de Souza, Nicole Felix, Josephine Harrington, Bruno Bezerra Lima

**Affiliations:** 1 Department of Medicine, University of Colorado Anschutz Medical Campus School of Medicine, Aurora, Colorado, USA; 2 Department of Medicine, Cleveland Clinic, Cleveland, Ohio, USA; 3 Department of Medicine, Universidade Federal do Ceara Faculdade de Medicina, Fortaleza, Brazil; 4 Department of Medicine, Federal University of Campina Grande, Campina Grande, Brazil; 5 Colorado Prevention Center, Aurora, Colorado, USA; 6 Department of Medicine, Cardiology Division, Vanderbilt University Medical Center, Nashville, Tennessee, USA

**Keywords:** Myocardial Infarction, Heart Failure, Pharmacology, Clinical, Meta-Analysis, Acute Coronary Syndrome

## Abstract

**Background:**

Left ventricular (LV) thrombus is a complication of myocardial infarction and dilated cardiomyopathy and is associated with a high thromboembolic risk. Although warfarin has traditionally been used, direct oral anticoagulants (DOACs) offer a more convenient alternative. With the addition of the RIVAWAR trial, we conducted an updated systematic review and meta-analysis to assess the efficacy and safety of DOACs compared with warfarin in patients with LV thrombus.

**Methods:**

A systematic search of electronic databases (PubMed, EMBASE, Cochrane and clinicaltrials.gov) from inception to April 2025 identified randomised clinical trials (RCTs) comparing DOACs with warfarin for the treatment of LV thrombus. The main outcome of interest was thrombus resolution at 3 months. Risk ratios (RRs) with 95% CIs were calculated using random-effects models.

**Results:**

Seven RCTs comprising 554 patients were included. Non-contrast transthoracic echocardiography was used for LV thrombus assessment in all RCTs. There was no difference between DOACs and warfarin in thrombus resolution at 3 months (RR 1.02; 95% CI 0.95 to 1.09), major adverse cardiovascular events (RR 0.50; 95% CI 0.16 to 1.54), all-cause mortality (RR 0.92; 95% CI 0.36 to 2.31), stroke/systemic embolism (RR 0.76; 95% CI 0.12 to 4.68), rehospitalisation (RR 1.36; 95% CI 0.47 to 3.94) or major bleeding (RR 0.54; 95% CI 0.20 to 1.48). Subgroup and sensitivity analyses confirmed the robustness of these results.

**Conclusions:**

DOACs demonstrated similar efficacy and safety to warfarin for LV thrombus management in this meta-analysis, supporting their use for the treatment of LV thrombus. However, large-scale RCTs with longer follow-up periods and using diagnostic modalities with higher sensitivity and specificity for detecting LV thrombus resolution are warranted to confirm these findings and clarify long-term outcomes.

**PROSPERO registration number:**

CRD420251023513.

**WHAT IS ALREADY KNOWN ON THIS TOPIC**

- The use of direct oral anticoagulants (DOACs) for managing left ventricular (LV) thrombus is supported by international society guidelines. However, these recommendations are based on small randomised controlled trials (RCTs) and meta-analyses that primarily include observational data and only a few RCTs.

**WHAT THIS STUDY ADDS**

- We conducted an updated systematic review and meta-analysis to assess the efficacy and safety of DOACs compared with warfarin in patients with LV thrombus, including the recently released large-scale RIVAWAR trial. This trial enrolled more patients than all previously published RCTs on DOACs for LV thrombus combined. The pooled analysis of seven RCTs showed no significant difference between DOACs and warfarin in thrombus resolution at 3 months, major adverse cardiovascular events, all-cause mortality, stroke/systemic embolism, rehospitalisation or major bleeding.

**HOW THIS STUDY MIGHT AFFECT RESEARCH, PRACTICE OR POLICY**

- By incorporating data from the RIVAWAR trial, this meta-analysis nearly triples the number of patients included in previous RCT-focused meta-analyses. Subgroup and sensitivity analyses consistently support DOACs as a viable alternative to warfarin for the treatment of LV thrombus with similar safety and efficacy profile.

## Introduction

 Left ventricular (LV) thrombus is a well-recognised complication of acute myocardial infarction (MI), particularly in anterior ST-segment elevation MI (STEMI), and is also frequently observed in patients with dilated cardiomyopathy.^1 2^ Reported incidence varies, ranging from approximately 6% in anterior STEMI cases to as high as 19% in patients with concomitant reduced LV function.^3 4^ Despite appropriate anticoagulation, LV thrombus remains a significant clinical concern due to its strong association with embolic events, including stroke and increased mortality.^1 5^

Direct oral anticoagulants (DOACs) have emerged as an attractive alternative to warfarin for treating LV thrombus, offering advantages such as fewer drug interactions, no dietary restrictions, a more predictable pharmacokinetic profile, reduced need for monitoring and greater ease of use in clinical practice.^6 7^ The 2023 European Society of Cardiology guidelines for the management of acute coronary syndromes already suggest considering DOACs for 3–6 months in patients with confirmed LV thrombus as an alternative to warfarin.^8^ However, this recommendation is based on small randomised clinical trials (RCTs) and meta-analyses that primarily include observational data and few RCTs.^9 10^

Here, we conducted an updated systematic review and meta-analysis to assess the efficacy and safety of DOACs compared with warfarin in patients with LV thrombus, including the large-scale recently released RIVAWAR trial. This trial enrolled a larger cohort than all previously published RCTs evaluating the use of DOACs for LV thrombus combined.^3 11 12^

## Materials and methods

This systematic review with meta-analysis was conducted in accordance with the Preferred Reporting Items for Systematic Reviews and Meta-Analyses guidelines and the Cochrane Handbook for Systematic Reviews of Interventions recommendations.^13 14^ As such, we prospectively registered the study protocol in the International Prospective Register for Systematic Reviews (PROSPERO) under protocol number CRD420251023513.

### Search strategy and data extraction

TAC and LMTdS systematically searched PubMed, Embase and Web of Science from inception to April 2025 with the following search terms: “left ventricle”, “left ventricular”, “LV”, “clot”, “thrombus”, “thrombi”, “thrombosis”, “intracardiac thrombus”, “DOAC”, “NOAC”, “direct anticoagulant”, “direct anticoagulants”, “direct oral anticoagulant”, “direct oral anticoagulants”, “oral anticoagulation”, “new oral anticoagulant”, “novel oral anticoagulant”, “novel oral anticoagulants”, “non-vitamin K antagonist oral anticoagulant”, “NVKOA”, “factor Xa inhibitor”, “inhibitor factor Xa”, “direct factor Xa inhibitor”, “direct factor Xa inhibitors”, “direct-acting oral anticoagulant”, “anticoagulant direct-acting oral”, “direct acting oral anticoagulant”, “direct-acting oral anticoagulants”, “direct acting oral anticoagulants”, “direct thrombin inhibitor”, “direct thrombin inhibitors”, “apixaban”, “dabigatran”, “edoxaban” and “rivaroxaban”, along with the Cochrane sensitive search for randomised studies. We applied no language or date restrictions, nor did we use any filters for the search in any database. The references from all included studies, previous systematic reviews and meta-analyses were also searched manually for any additional studies.^15^ Two authors (TAC and LMTdS) independently selected the studies following predefined search criteria and quality assessment.

### Eligibility criteria

Inclusion in this meta-analysis was restricted to studies that met all of the following eligibility criteria: (1) RCTs or *post-hoc* analyses of RCTs; (2) comparing a DOAC (eg, apixaban, dabigatran, edoxaban or rivaroxaban) with warfarin; (3) enrolling persons with proven LV thrombus, with diagnosis based on the definitions and imaging criteria reported in the original studies and (4) reporting outcomes of interest. We excluded studies with no control groups.

### Endpoints

The main outcome of interest was LV thrombus resolution. Secondary outcomes included major adverse cardiac events (MACE: defined as a composite of death from cardiovascular causes, MI or stroke or systemic emboli), all-cause mortality (ACM), stroke or systemic emboli, need for rehospitalisation and major bleeding.

### Quality assessment

Quality assessment of RCTs and their post-hoc analyses was performed by two authors (GCLC and BLLdS) using the Cochrane Collaboration’s tool for assessing risk of bias in randomised trials version 2 (RoB 2).^16^

### Statistical analysis

We pooled the absolute (crude) number of events from individual studies to estimate risk ratios (RRs) with 95% CIs for binary endpoints. P values <0.05 were deemed significant for treatment effects. We assessed the heterogeneity of included studies using Cochran’s Q test and the Thompson I² value, with p values <0.10 deemed significant for the Cochran’s Q. I² values were interpreted as low (<25%), moderate (25% to <50%), substantial (50% to <75%) and considerable heterogeneity (≥75%).^17^ We applied a restricted maximum likelihood random-effects model for all outcomes to account for methodological and demographic heterogeneity across included studies, as per Cochrane recommendations.^14 18^

To assess the robustness of our findings, we performed several sensitivity analyses. We first conducted leave-one-out sensitivity analyses to investigate the influence of individual studies in the pooled analyses and identify potential outliers that could influence the observed overall effect size. We then applied a treatment-arm continuity correction (TACC) as an alternative to the standard 0.5 continuity correction for zero-event studies. Next, we performed meta-analyses using the Hartung-Knapp-Sidik-Jonkman (HKSJ) method to improve type I error control in small samples. For outcomes with very low event rates, we estimated the risk difference (RD) with 95% prediction intervals to illustrate the expected dispersion in future studies. Finally, we explored generalised linear mixed models (GLMM) for rare or zero-event outcomes.

We performed subgroup analyses by stratifying patients according to LV thrombus aetiology (post-MI vs non-specified), type of DOAC used, duration of triple therapy following MI and risk of bias assessment for outcomes where more than two included RCTs contributed events to the pooled analysis.

To further assess robustness and control for random errors due to repetitive testing, we performed a trial sequential analysis (TSA) for the main outcome, using the Trial Sequential Analysis computer programme V.0.9.5.10 Beta (The Copenhagen Trial Unit, Centre for Clinical Intervention Research, The Capital Region, Copenhagen University Hospital, Rigshospitalet, 2021). A two-sided testing approach was used, with a type I error of 5% and a type II error of 20% (corresponding to 80% power). Both conventional and alpha-spending monitoring boundaries were constructed for comparisons between DOACs and warfarin. A random-effects model with 95% CIs was applied to account for potential heterogeneity. The cumulative Z-curve was plotted against the number of accrued events, as event size was used as the information axis, which is appropriate for dichotomous outcomes. Significance thresholds were adjusted using the O’Brien-Fleming alpha-spending function. Additionally, the required information size (RIS), defined as the total number of events needed to reliably accept or reject the intervention effect, was calculated. To handle trials with zero events, a continuity correction of 0.5 was applied and trials with zero events in both arms were included in the analysis.

This meta-analysis was performed using R software V.4.4.2 (R Foundation for Statistical Computing, Vienna, Austria, 2024) using the BiasedUrn, dmetar, meta, metafor and readxl packages and Review Manager V.5.4.1 (Nordic Cochrane Centre, The Cochrane Collaboration, Copenhagen, Denmark, 2020).

## Results

### Study selection and characteristics

As shown in online supplemental figure 1, our initial search identified 745 studies. After removing duplicate records, 540 studies remained and were assessed based on our inclusion and exclusion criteria. Ultimately, we included a total of seven RCTs, comprising 554 patients.^310^,^12 19^

Of the included studies, five enrolled only patients with post-MI LV thrombus, comprising 444 individuals and representing 80% of the overall study population.^311 12 20^,^22^ Two studies did not specify the aetiology of LV thrombus; however, Abdelnabi *et al* and Isa *et al* reported ischaemic cardiomyopathy in 78.5% and 62.9% of their respective cohorts.^10 19^ Abdelnabi *et al* reported that 53.1% of patients were on triple therapy, while all postangioplasty patients in the Isa *et al* study received triple therapy.^10 19^ However, neither study specified the duration of triple therapy nor stratified clinical outcomes based on its use.^10 19^

Overall, 58.1% of participants (n=322) received a DOAC-based regimen, with 59 patients (18.3%) treated with apixaban and 263 (81.7%) with rivaroxaban. Detailed baseline study characteristics are shown in online supplemental table 1.

### Pooled analysis of all studies

#### Main outcome

**Figure 1:**
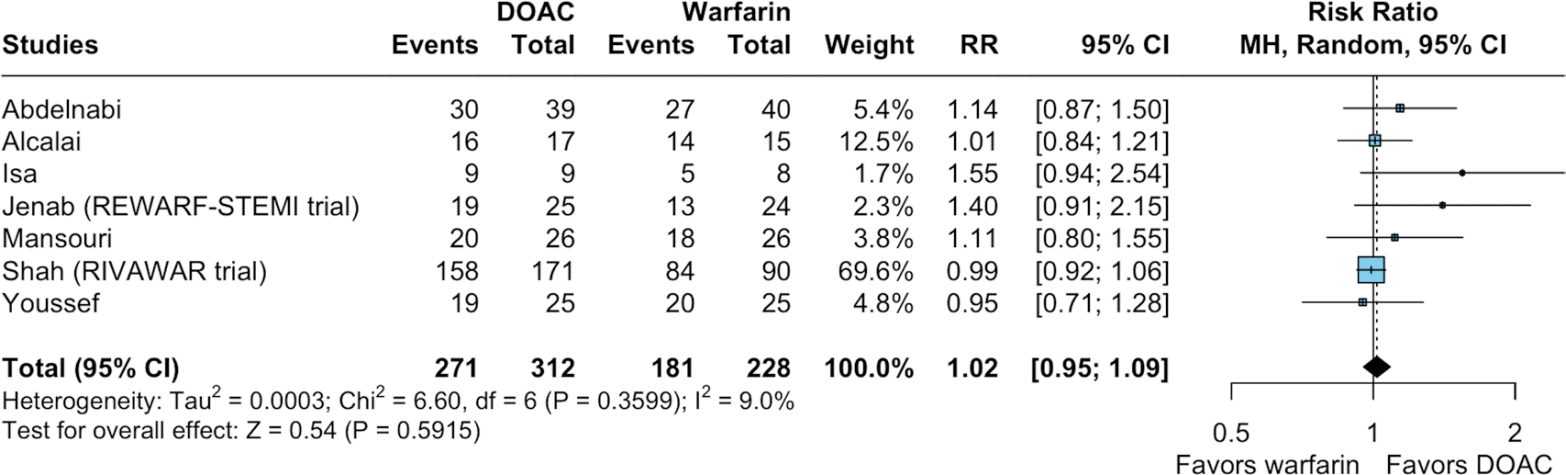
Forest plots for left ventricular (LV) thrombus resolution at 3 months. The use of direct oral anticoagulants (DOACs) therapy had comparable relative risk of LV thrombus resolution at 3 months compared with warfarin. CI, Confidence Interval; MH, Mantel–Haenszel; RR, risk ratio; STEMI, ST-segment elevation myocardial infarction.

Seven studies evaluated thrombus resolution at 3 months. Overall, there was no significant difference between the DOAC-based and warfarin-based regimens (RR 1.02; 95% CI 0.95 to 1.09; p=0.591; I²=9%; figure 1).

#### Sensitivity analyses for the main outcome

Subgroup sensitivity analyses for the main outcome showed no significant treatment interactions (test for subgroup difference with p≥0.10) based on thrombus aetiology (post-MI vs non-specified), DOAC used, duration of triple therapy and risk of bias assessment (online supplemental figure 2).

A leave-one-out sensitivity analysis was conducted, sequentially omitting each study from the meta-analysis to identify potential outliers that could influence the observed overall effect size and heterogeneity (online supplemental figure 3). The effect size remained consistent across all iterations, ranging from 1.01 to 1.08, with all estimates showing no statistical significance. Heterogeneity remained stable and was considered low (I²=0 to 24.1%). These findings confirm the robustness of our results, indicating that no single study has a disproportionate effect on the overall conclusions.

Two studies assessed thrombus resolution at 6 months, and the measured effect remained consistent (RR 1.0; 95% CI 0.89 to 1.13; p=0.297; I²=8.2%; online supplemental figure 4).

#### Efficacy outcomes

**Figure 2:**
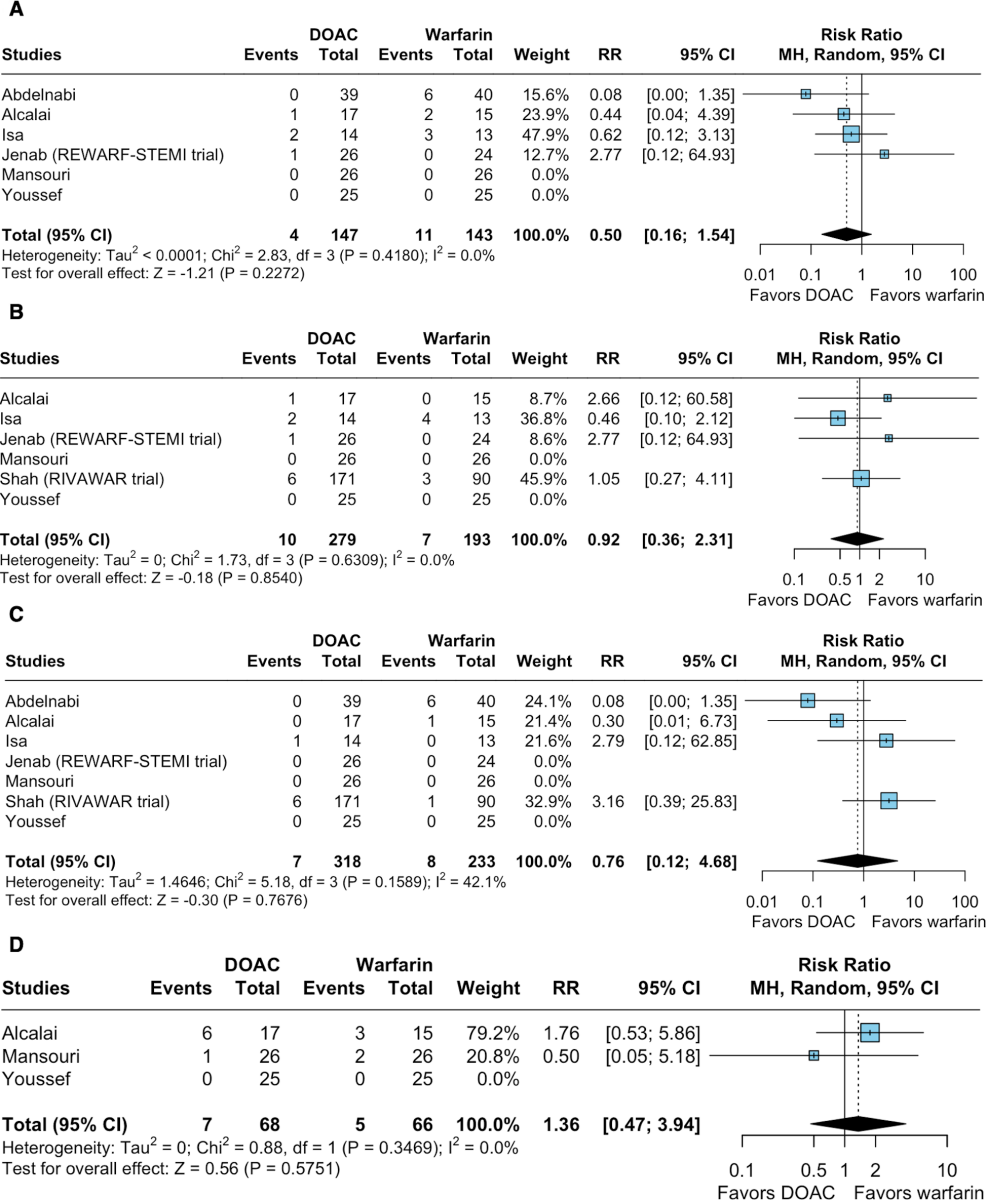
Forest plots for efficacy endpoints compared with warfarin, the use of direct oral anticoagulants (DOACs) therapy had comparable relative risk of (**A**) major adverse cardiac events (MACE), (**B**) all-cause mortality, (**C**) stroke or systemic emboli events and (**D**) rehospitalisation. CI, Confidence Interval; MH, Mantel–Haenszel; RR, risk ratio; STEMI, ST-segment elevation myocardial infarction.

There was no significant difference between the groups regarding MACE (RR 0.50; 95% CI 0.16 to 1.54; p=0.227; I²=0%; figure 2A), ACM (RR 0.92; 95% CI 0.36 to 2.31; p=0.854; I²=0%; figure 2B), stroke or systemic emboli (RR 0.76; 95% CI 0.12 to 4.68; p=0.768; I²=42.1%; figure 2C) and rehospitalisation (RR 1.36; 95% CI 0.47 to 3.94; p=0.575; I²=0%; figure 2D).

#### Sensitivity analyses for efficacy outcomes

There were no significant treatment interactions (test for subgroup difference with p*≥*0.10) based on thrombus aetiology (post-MI vs non-specified), DOAC used, duration of triple therapy and risk of bias assessment for MACE, ACM, stroke or systemic emboli (online supplemental figure 5). However, a significant interaction was identified between risk-of-bias assessment and stroke or systemic emboli (online supplemental figure 5.3). The outcome of rehospitalisation had only two studies contributing events; therefore, sensitivity analysis was not conducted for this outcome.

In leave-one-out sensitivity analyses (online supplemental figure 6), the effect size remained consistent across all iterations for MACE, ACM and stroke or systemic emboli. Heterogeneity decreased for stroke or systemic emboli after omitting the study by Abdelnabi *et al* (RR 1.75; 95% CI 0.38 to 8.01; I²=0%).

#### Safety outcomes

**Figure 3:**
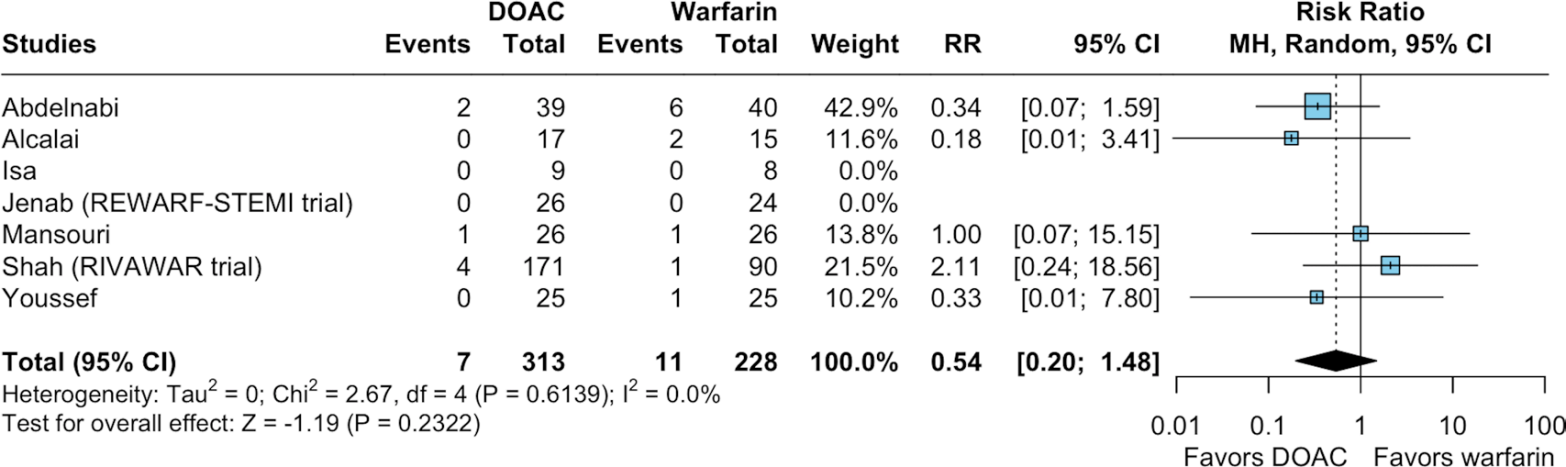
Forest plots for safety endpoints, Compared with warfarin, the use of direct oral anticoagulants (DOACs) therapy had comparable relative risk of major bleeding events. CI, Confidence Interval; MH, Mantel–Haenszel; RR, risk ratio; STEMI, ST-segment elevation myocardial infarction.

There were no significant differences between the groups in the occurrence of major bleeding (RR 0.54; 95% CI 0.20 to 1.48; p=0.232; I²=0%; figure 3), as defined in the included studies. Of note, major bleeding was defined according to International Society on Thrombosis and Haemostasis (ISTH) criteria in four trials (Alcalai *et al*., No-LVT, REWARF-STEMI and RIVAWAR), while Youssef *et al* used the Bleeding Academic Research Consortium (BARC) definition, but reported one episode of major bleeding according to ISTH criteria.^3 10 11 21 22^ Isa *et al* and Mansouri *et al* did not specify a definition for bleeding; however, neither study contributed any events to the major bleeding outcome.^19 20^

#### Sensitivity analyses for safety outcomes

There were no significant treatment interactions (test for subgroup difference with p*≥*0.10) for the outcome of major bleeding based on thrombus aetiology (post-MI vs non-specified), DOAC used, duration of triple therapy and risk of bias assessment for major bleeding (online supplemental figure 7). Leave-one-out analyses showed that the effect size and heterogeneity remained consistent across all iterations for major bleeding events (online supplemental figure 8).

Overall, the sensitivity analyses demonstrated that the pooled estimates were consistent across different methods (online supplemental table 2). RRs obtained with TACC and HKSJ adjustments were similar to the primary random-effects results. For rare events, RD estimates and 95% prediction intervals indicated limited variability across studies. GLMM analyses were successfully performed for most outcomes, although for some rare events, the model did not converge, which is explicitly noted. Collectively, these analyses support the robustness of the primary findings.

### Trial sequential analysis

**Figure 4:**
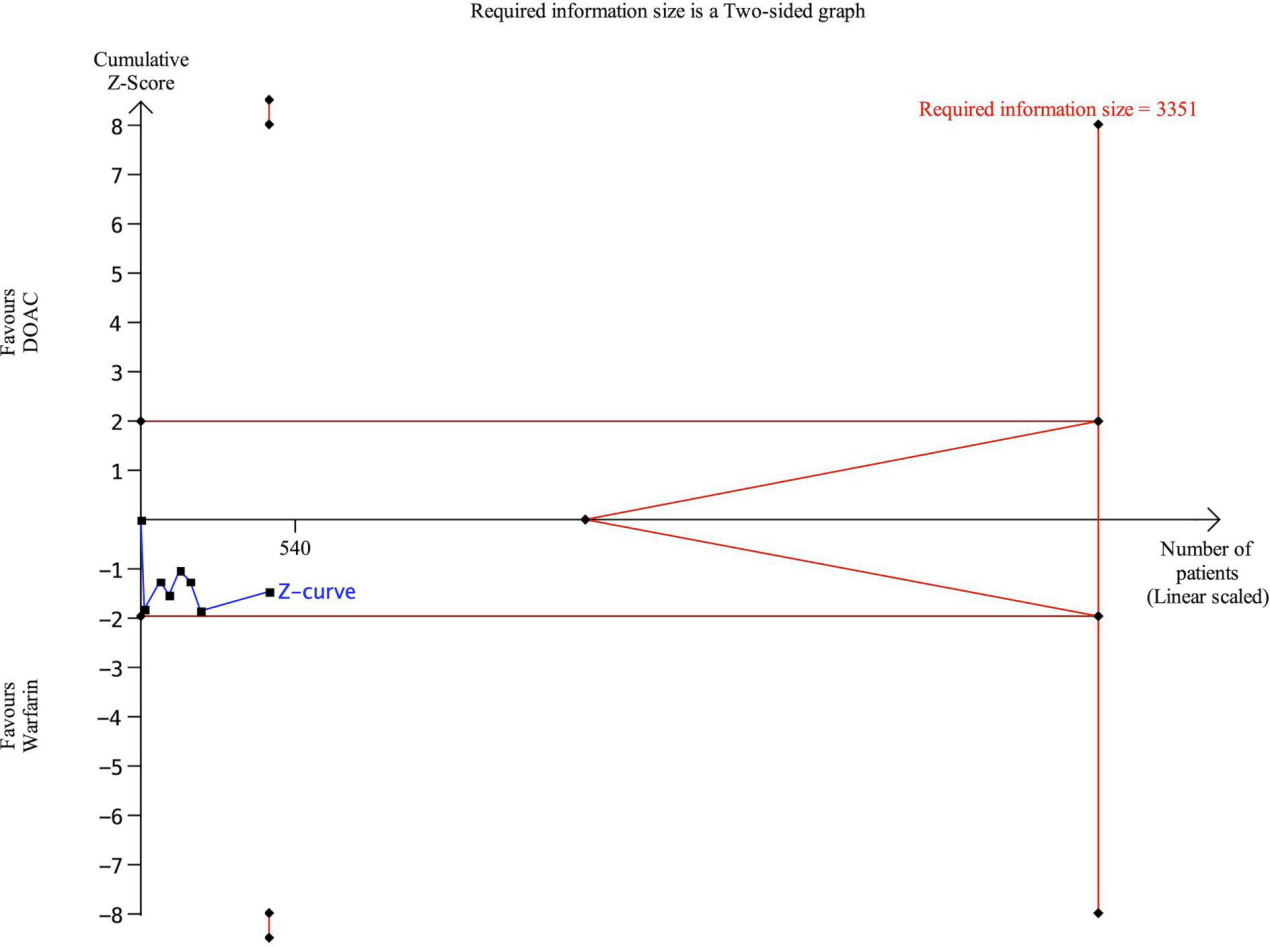
Trial sequential analyses. Trial sequential analyses for the outcome of left ventricular thrombus resolution at 3 months. DOAC, direct oral anticoagulants.

We conducted a TSA focusing on the main outcome, thrombus resolution at 3 months. TSA demonstrated that the cumulative Z-curves for thrombus resolution at 3 months remained within the conventional significance boundaries. The Z-curve did not cross trial sequential monitoring or futility boundaries and did not reach the RIS (3351 patients) (figure 4).

Given that the cumulative Z-curve did not cross the futility boundary or reach the RIS for this outcome, the current evidence may remain statistically inconclusive. Although we hypothesised that no meaningful difference exists between DOACs and warfarin, we cannot rule out the possibility of our analysis being underpowered.

### Risk of bias assessment

The results of the risk of bias assessment are shown in online supplemental figure 9. Studies by Abdelnabi *et al* and Youssef *et al* were judged to raise some concerns regarding potential bias arising from the randomisation process.^10 21^ In the study by Abdelnabi *et al*, this was due to the absence of information on allocation sequence concealment and a lack of details comparing baseline characteristics.^10^ Although a statement was provided, the absence of supporting evidence limits the ability to assess its credibility. Youssef *et al* presented significant differences between groups in terms of hospital stay duration and the number of study-related visits.^21^

Studies by Abdelnabi *et al* and Youssef *et al* were also assessed as raising some concerns regarding deviations from intended interventions.^10 21^ Abdelnabi *et al* failed to report whether any deviations from the intended interventions occurred.^10^ Youssef *et al* reported a violation of good clinical practice during the consent process, with the treating physician reassigning patients to a different study group on one clinical site.^21^

### Quality assessment

The Grading of Recommendations Assessment, Development and Evaluation (GRADE) assessment and summary of findings can be found in online supplemental table 3.

Overall, DOACs appear to be at least as effective as warfarin for thrombus resolution and may be associated with fewer adverse cardiovascular events and bleeding complications, with moderate-certainty evidence supporting these findings according to the GRADE assessment. The moderate certainty of evidence for these outcomes was primarily driven by imprecision due to: (1) TSA findings showing that the cumulative Z-curve did not cross the monitoring or futility boundaries and that the RIS (3351 patients) was not reached; (2) the use of non-contrast transthoracic echocardiography (TTE) as the diagnostic modality to assess LV thrombus resolution in all included RCTs and (3) the wide CIs observed for several outcomes of interest.

## Discussion

In this updated systematic review and meta-analysis of seven RCTs, we found that the use of DOACs in patients with LV thrombus was associated with comparable rates of thrombus resolution at 3 months, when assessed by non-contrast TTE, as well as similar incidences of MACE, ACM, stroke or systemic embolism, rehospitalisation and major bleeding events compared with warfarin. These findings remained consistent across subgroup analyses stratified by thrombus aetiology (post-MI vs non-specified), type of DOAC used (rivaroxaban vs apixaban), duration of triple therapy and risk of bias. The moderate certainty of evidence, as assessed by GRADE, underscores the need for further well-powered RCTs to confirm these results using diagnostic modalities with greater sensitivity and specificity for detecting LV thrombus resolution (eg, cardiac MRI [CMR] or contrast-enhanced TTE).

DOACs have been shown to be an effective anticoagulation strategy for the treatment of deep vein thrombosis, as well as for the prevention of stroke and systemic embolic events in patients with atrial fibrillation across diverse populations.^6 23^ In individuals with LV thrombus, the annual risk of stroke or systemic embolism is estimated at 10–15% if left untreated.^7^ Given their favourable pharmacological profile, DOACs have emerged as an appealing alternative to warfarin for managing LV thrombus, offering ease of use, predictable effects and fewer requirements for monitoring or concern for drug interactions.^6^

Previous meta-analyses have highlighted the potential benefit of DOACs for treating patients with LV thrombus, which is consistent with our findings.^3 9 24 25^ However, these studies were limited by the predominance of observational data, largely due to small sample sizes in earlier trials, which may introduce confounding and result in inconsistencies. For example, Haller *et al* reported a lower risk of major bleeding and ACM with DOAC use,^25^ whereas Pasqualotto *et al*, in their subsequent meta-analysis of 29 studies, found no significant differences between DOACs and warfarin for bleeding outcomes.^24^

In contrast, our meta-analysis only included RCTs and incorporated the recently presented RIVAWAR trial, which contributed data from 261 patients (158 in the DOAC arm and 84 in the warfarin arm).^11 12^ This addition increased our sample size to almost three-fold the number of patients included in previous meta-analyses focused exclusively on RCTs.^3^ Although several of our outcomes overlapped with those of the 2024 meta-analyses, important methodological distinctions should be emphasised. Pasqualotto *et al* defined bleeding broadly (any, clinically relevant, minor or major events), whereas Haller *et al* used a composite bleeding endpoint—differences that may partly explain the variation in results.^24 25^ Moreover, while Haller *et al* provided details on imaging modalities used for thrombus follow-up, Pasqualotto *et al* did not specify preferred imaging methods.^24 25^ Finally, our included RCTs were relatively homogeneous, with follow-up durations ranging from 3 months to 6 months, whereas both 2024 meta-analyses combined RCTs and observational studies with wider follow-up windows (from 3 months to >1 year), which might have impacted their findings to some extent.^24 25^

Our analysis was not designed to determine the optimal duration of anticoagulation therapy for LV thrombus. However, the high incidence of thrombus resolution at 3 months observed in the pooled analysis (86.8% in the DOAC group and 79.4% in the warfarin group) supports the current recommendations made in the American Heart Association’s Scientific Statement, which advocates for repeat imaging at 3 months using the same or a more advanced imaging modality as initially used for diagnosis.^7^ If thrombus resolution is confirmed, it appears reasonable to discontinue anticoagulation at that point, but a shared decision-making approach should be applied as to whether anticoagulation should be continued indefinitely.^7^

Our study was primarily limited by the fact that all included trials relied on non-contrast TTE for the diagnosis and follow-up of LV thrombus. While TTE is a validated and widely used modality, it is less sensitive than CMR or contrast-enhanced TTE and may therefore underestimate thrombus prevalence and overestimate resolution rates.^4^ Although this limitation likely affected both treatment groups equally, it may have influenced the absolute rates of thrombus detection and resolution, which may artificially equalise regimens by missing residual thrombi, thereby impacting the interpretation of clinical efficacy (online supplemental figure 10). Sensitivity analyses restricted to studies employing CMR or contrast-enhanced TTE were not possible due to the unavailability of such data in the existing trials. Future trials should incorporate CMR or contrast-enhanced TTE to improve diagnostic accuracy and outcome assessment. Moreover, the relatively short duration of follow-up across most studies (typically limited to 3 months) may also limit long-term outcome interpretation.

Second, this study is limited by the lack of individual participant-level data, which restricted our ability to conduct more detailed subgroup analyses, such as stratification by ejection fraction or by the specific type of dual antiplatelet therapy used during the triple therapy period. Notably, the duration of triple therapy varied across studies (ranging from 7 days to 90 days), which could represent a potential residual confounder for the major bleeding and embolic outcomes. To address this, we conducted a sensitivity analysis comparing shorter (<30 days) versus longer (≥30 days) durations of triple therapy, which showed no significant treatment interaction between the groups. Additionally, two studies did not report the underlying aetiology of LV thrombus, further limiting subgroup analyses. Two studies raised concerns related to the randomisation process and deviations from the intended interventions; however, sensitivity analyses stratified by the risk of bias assessment showed no significant change in the estimated treatment effects.

In conclusion, this systematic review and meta-analysis provides the most comprehensive and up-to-date synthesis of all available RCTs, suggesting that DOACs may be considered reasonable alternatives to warfarin for LVT treatment, given their comparable efficacy and safety when careful imaging follow-up is ensured and antiplatelet regimens are thoughtfully minimised. Ongoing RCTs (eg, NCT06013020, NCT05892042 and NCT05705089) are needed to confirm these findings and further evaluate the impact of DOACs on clinical outcomes in this population, ideally using diagnostic modalities with higher sensitivity and specificity for detecting LV thrombus resolution and incorporating longer follow-up durations.

## Supplementary material

10.1136/openhrt-2025-003542 online supplemental file 1

## Data Availability

Data are available in a public, open access repository. All data relevant to the study are included in the article or uploaded as supplementary information.
