# Supplementary material for: Efficacy and safety of direct oral anticoagulants versus warfarin in patients with a left ventricular thrombus: an updated systematic review and meta-analysis of randomised controlled trials
Source: Open Heart. 2025 Nov 19;12(2):e003542. doi: 10.1136/openhrt-2025-003542 (PMC12636967; doi:10.1136/openhrt-2025-003542)
Supplement: online supplemental file 1 [file openhrt-12-2-s001.docx]

**Supplementary Material**

**Supplementary Figure 1.** PRISMA flow diagram of study screening and selection.


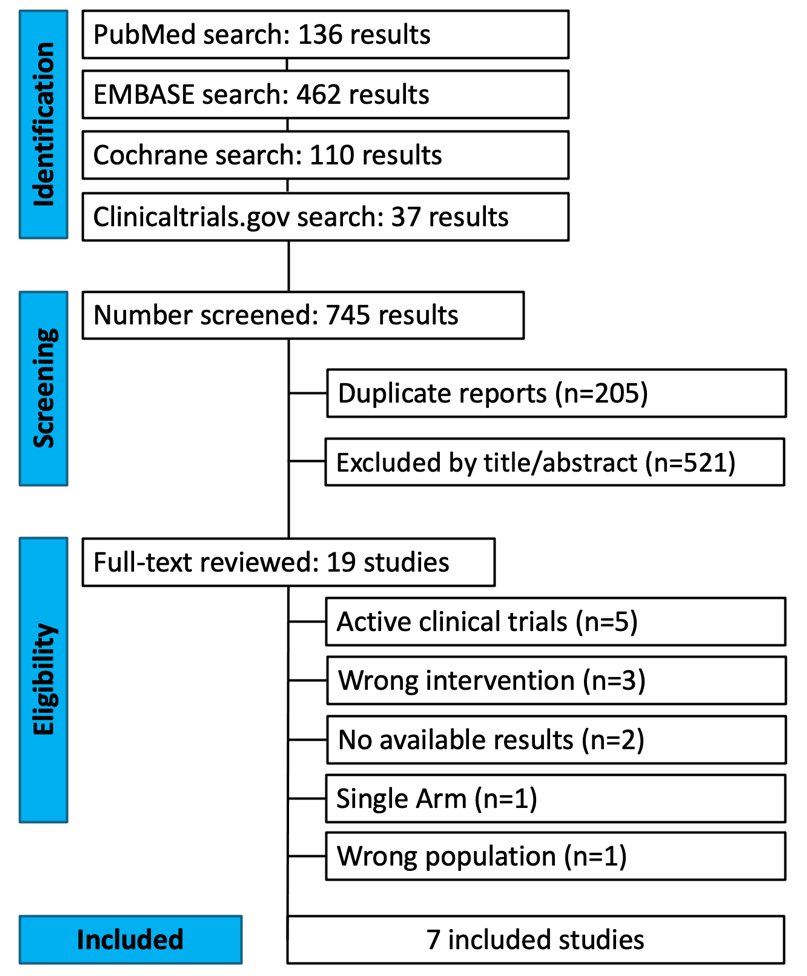


**Supplementary Figure 2.** Forest plot of subgroup sensitivity analyses for the outcome of left ventricular (LV) thrombus resolution at 3 months based on **(A)** thrombus etiology (non-specified *vs* post-MI), **(B)** DOAC used, **(C)** duration of triple therapy, and **(D)** risk of bias assessment. Abbreviations: **CI:** confidence interval; **DOAC**: direct oral anticoagulants; **MH:** Mantel-Haenszel; **RR**: risk ratio.


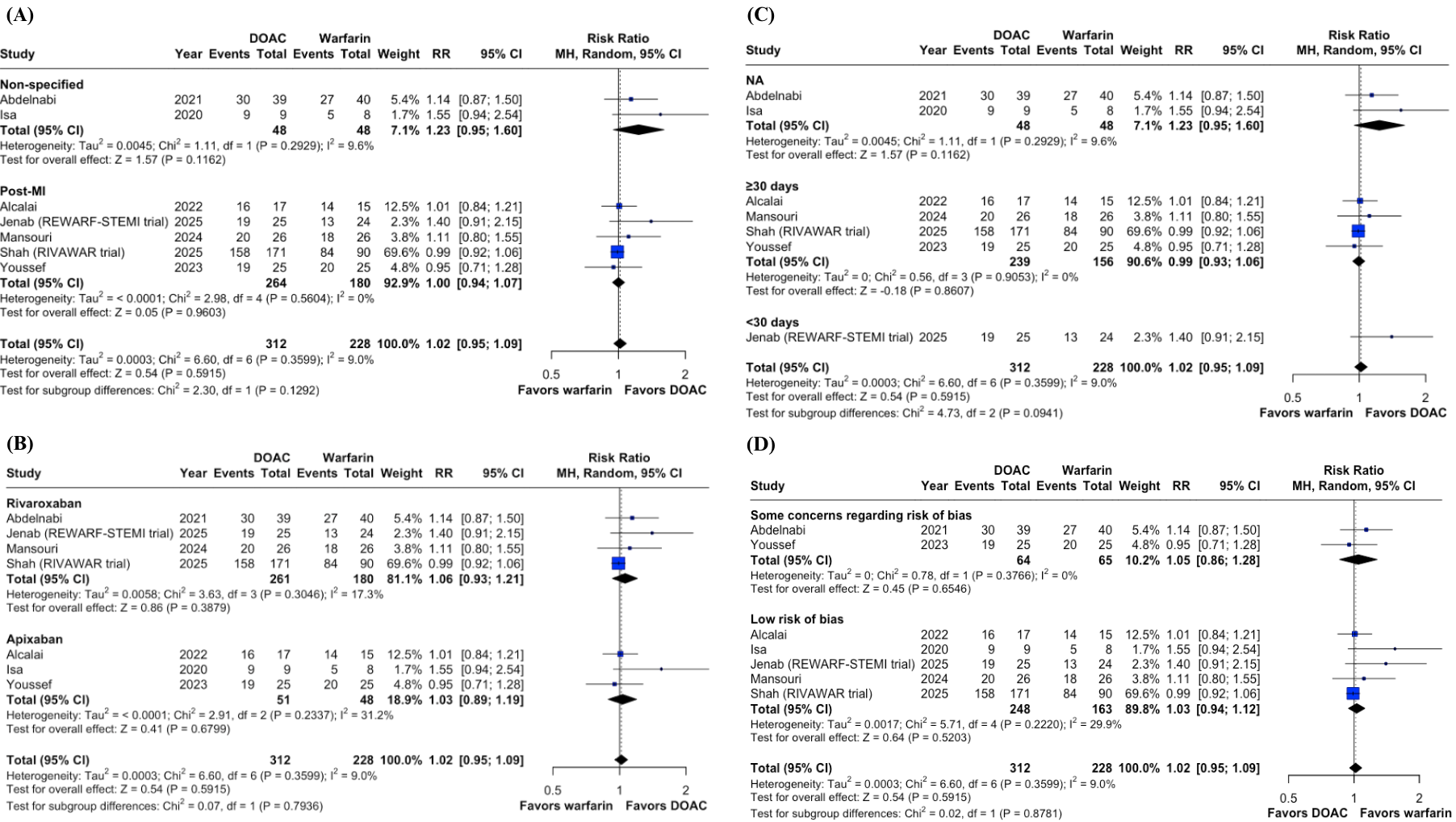


**Supplementary Figure 3.** Forest plot of leave-one-out sensitivity analyses for the outcome of left ventricular (LV) thrombus resolution at 3 months.


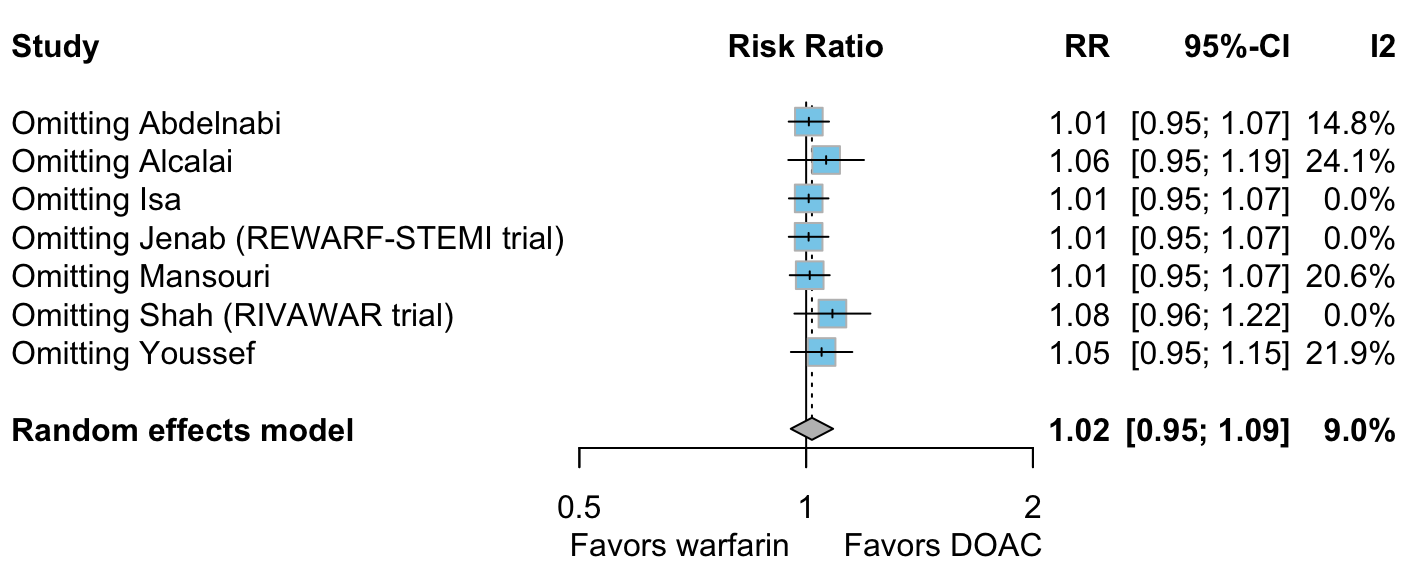


**Supplementary Figure 4.** Forest plot of sensitivity analysis evaluating the resolution of left ventricular (LV) thrombus at 6 months in the available studies. Abbreviations: **CI:** confidence interval; **DOAC**: direct oral anticoagulants; **MH:** Mantel-Haenszel; **RR**: risk ratio.


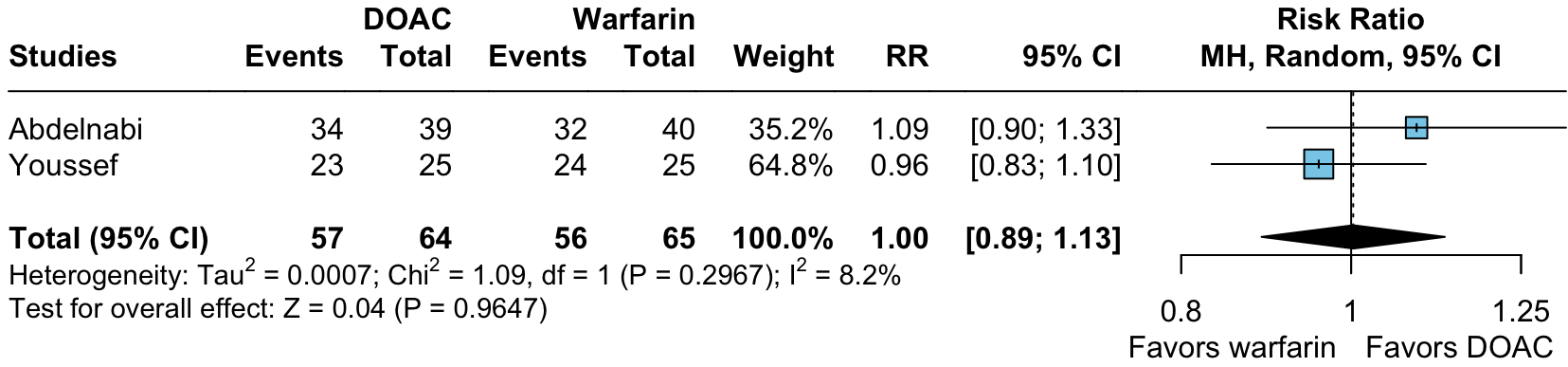


**Supplementary Figure 5.** Forest plot of subgroup sensitivity analyses for the outcomes of 5.1 Major Adverse Cardiovascular Events (MACE), 5.2 All-cause mortality (ACM), and 5.3 stroke or systemic emboli (SSE) based on **(A)** thrombus etiology (non-specified *vs* post-myocardial infarction [MI]), **(B)** DOAC used, **(C)** duration of triple therapy, and **(D)** risk of bias assessment. Abbreviations: **CI:** confidence interval; **DOAC**: direct oral anticoagulants; **MH:** Mantel-Haenszel; **RR**: risk ratio.

**5.1**. **MACE**

**
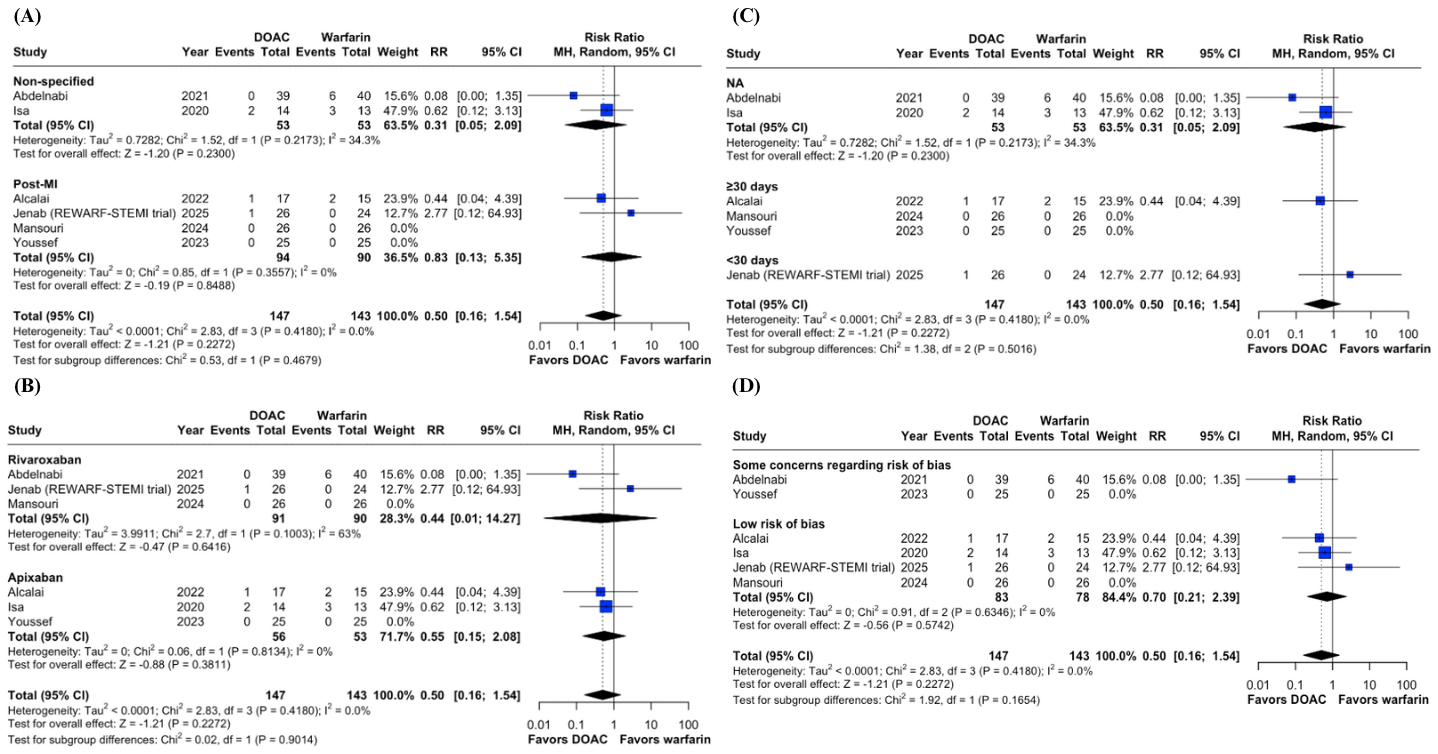
**

**5.2 ACM**


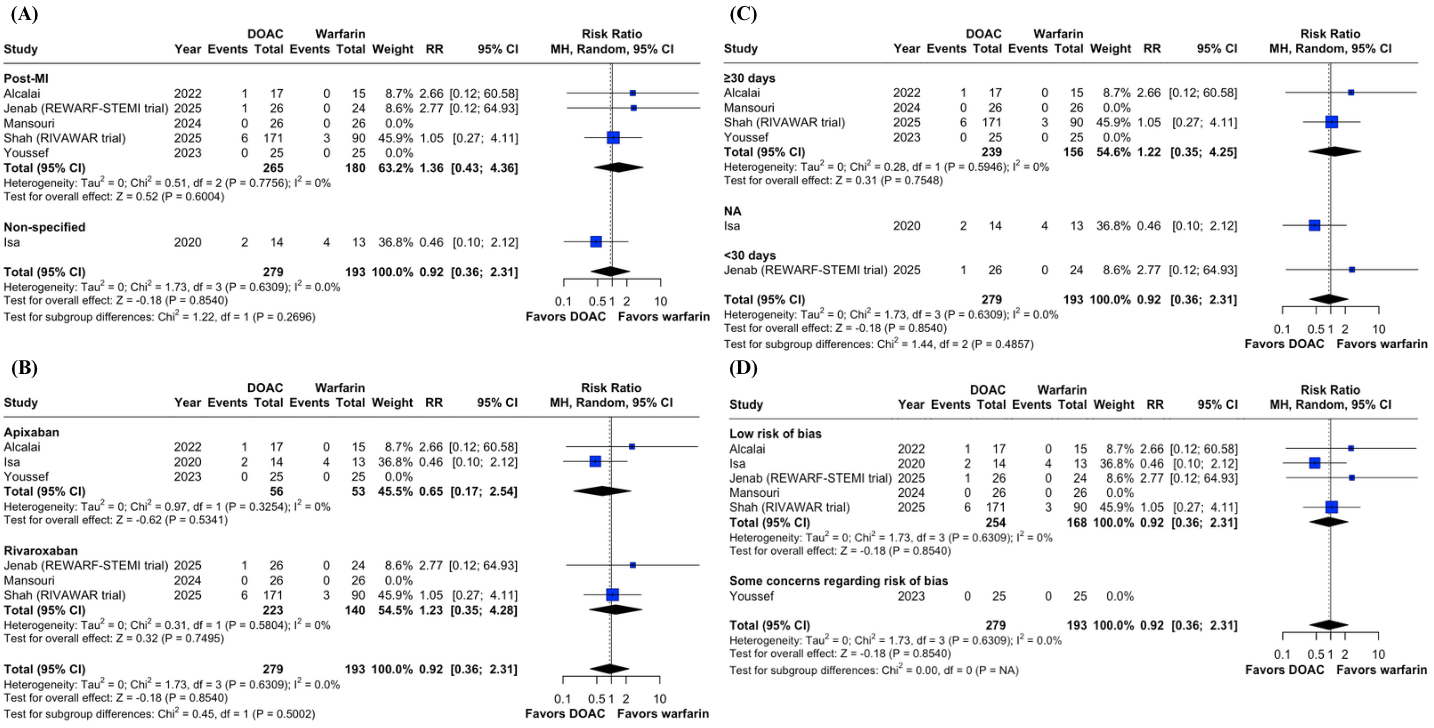


**5.3 SSE**


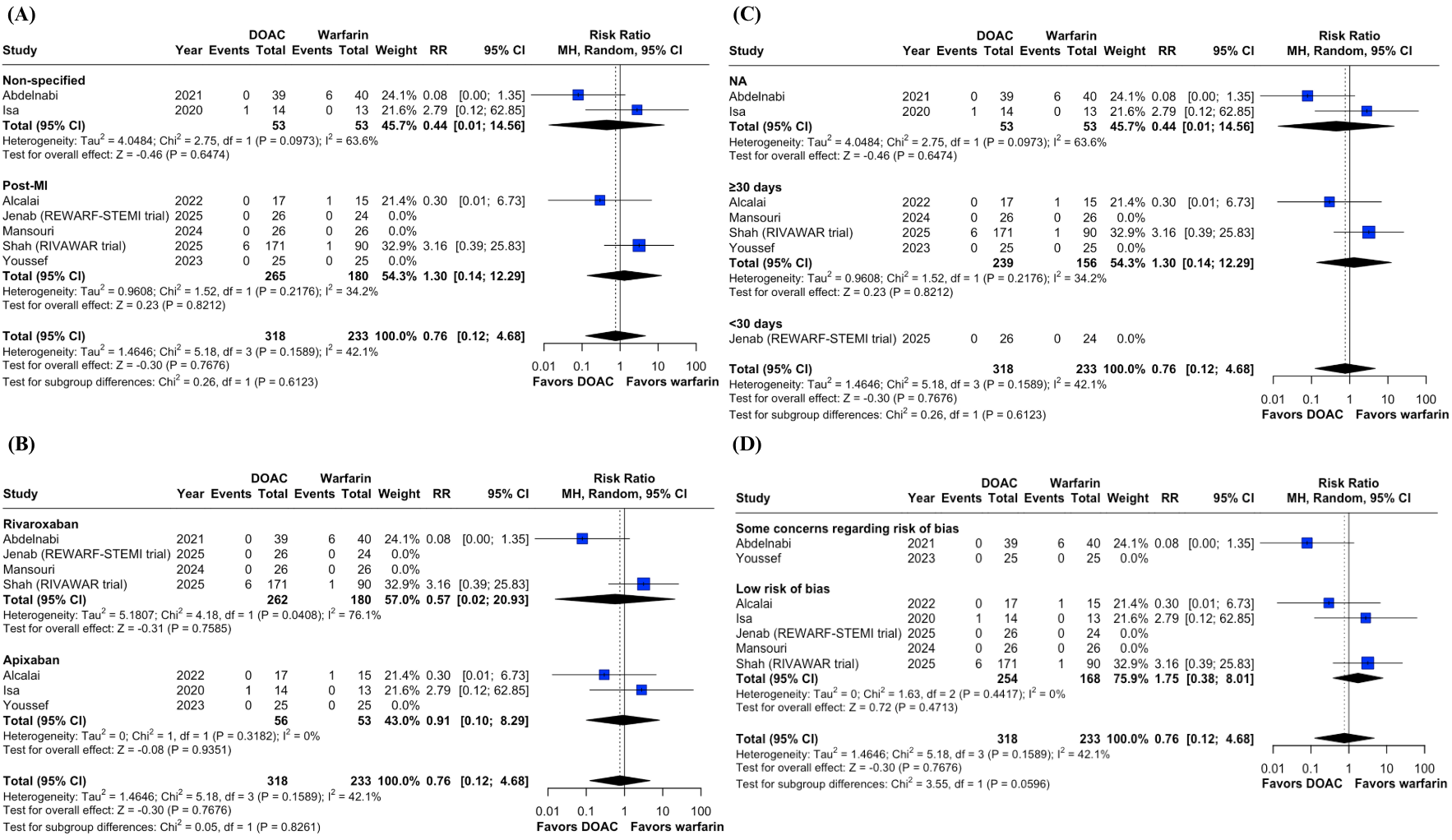


**Supplementary Figure 6.** Forest plot of leave-one-out sensitivity analyses for the outcomes of **(A)** Major Adverse Cardiovascular Events (MACE), **(B)** All-cause mortality (ACM), and **(C)** stroke or systemic emboli.


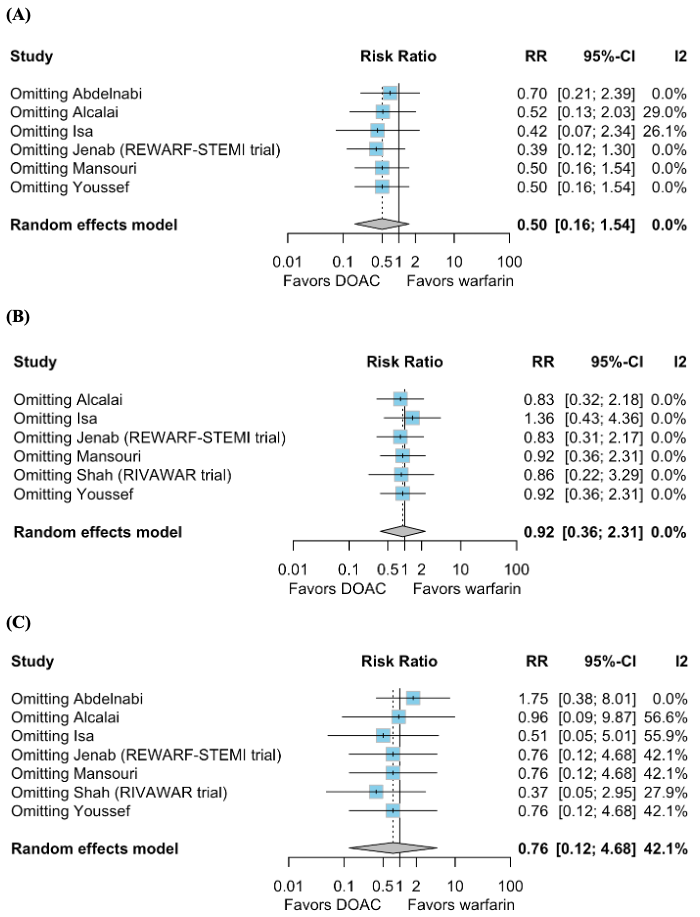


**Supplementary Figure 7.** Forest plot of subgroup sensitivity analyses for the outcome of major bleeding based on **(A)** thrombus etiology (non-specified *vs* post-myocardial infarction [MI]), **(B)** DOAC used, **(C)** duration of triple therapy, and **(D)** risk of bias assessment. Abbreviations: **CI:** confidence interval; **DOAC**: direct oral anticoagulants; **MH:** Mantel-Haenszel; **RR**: risk ratio.


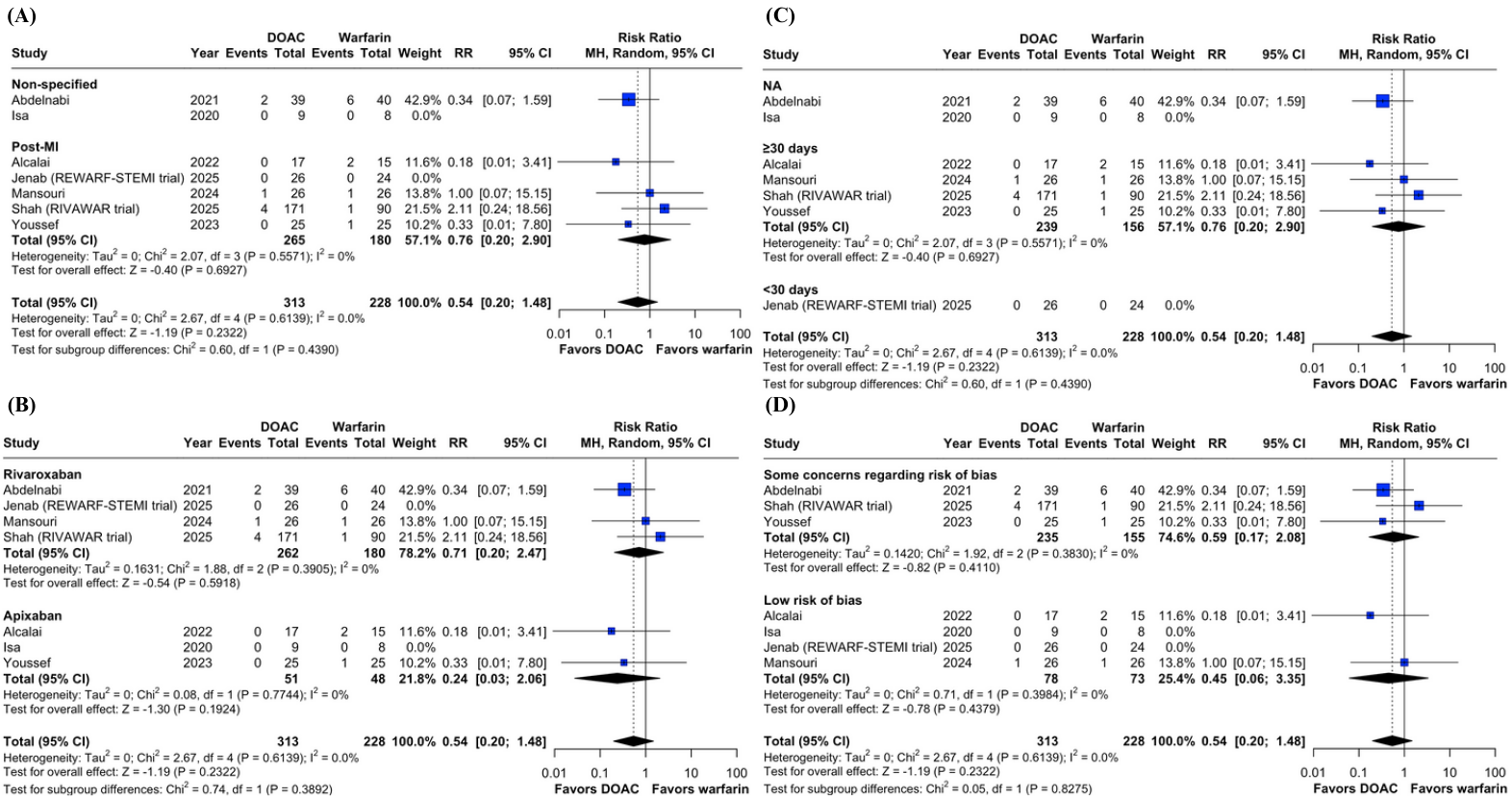


**Supplementary Figure 8.** Forest plot of leave-one-out sensitivity analyses for the outcome of major bleeding.

**
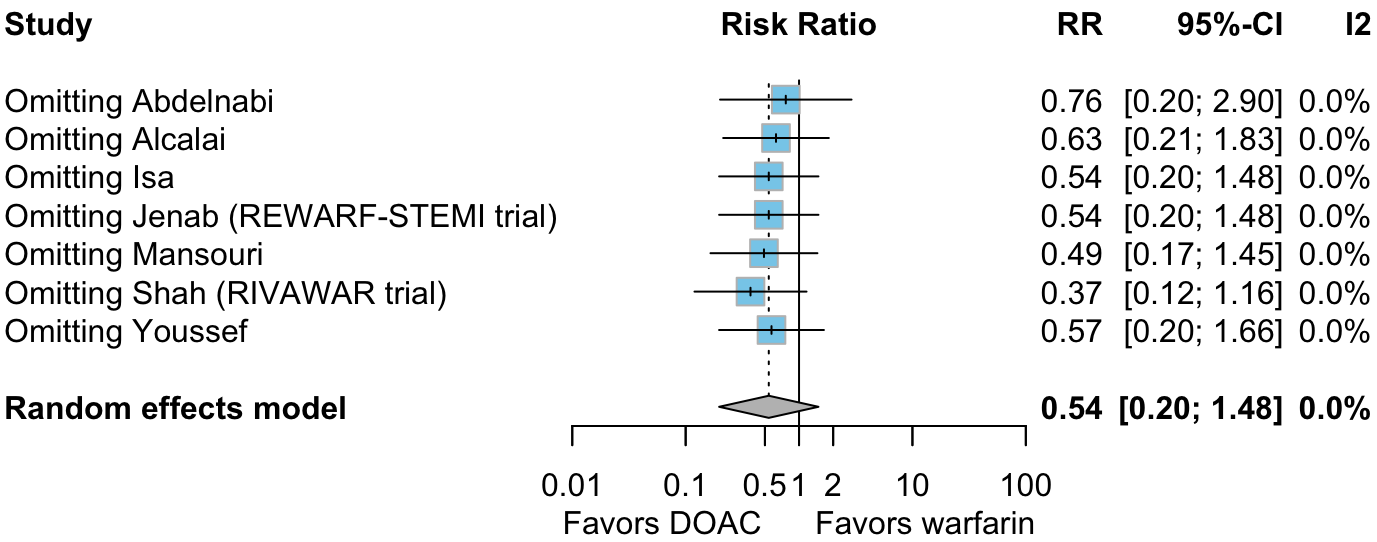
**

**Supplementary Figure 9**. Risk of bias assessment of individual studies.

**
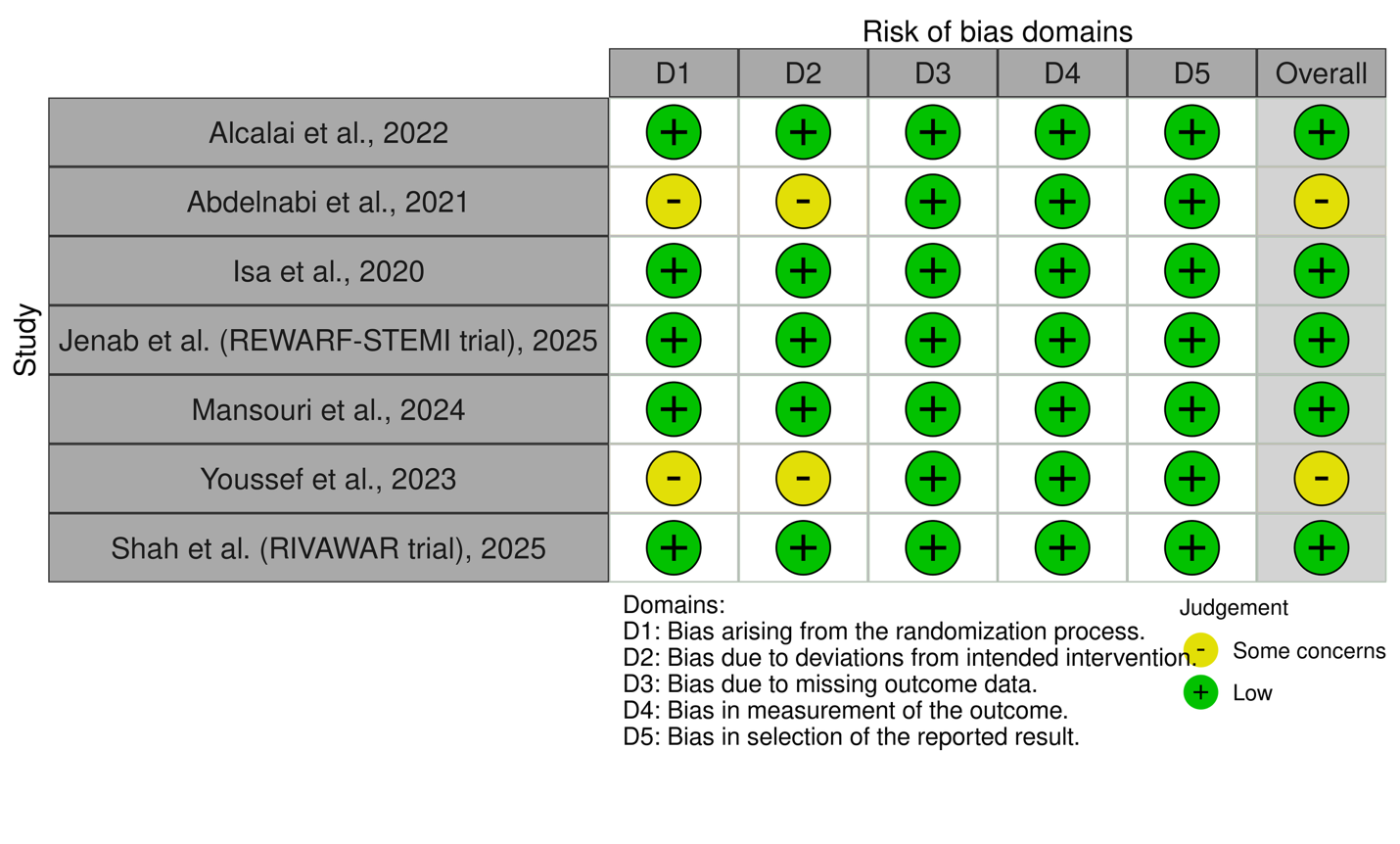
**

**Supplementary Figure 10. Directed acyclic graph (DAG) illustrating the potential measurement bias pathway associated with the use of non-contrast TTE in assessing LV thrombus resolution.**

**
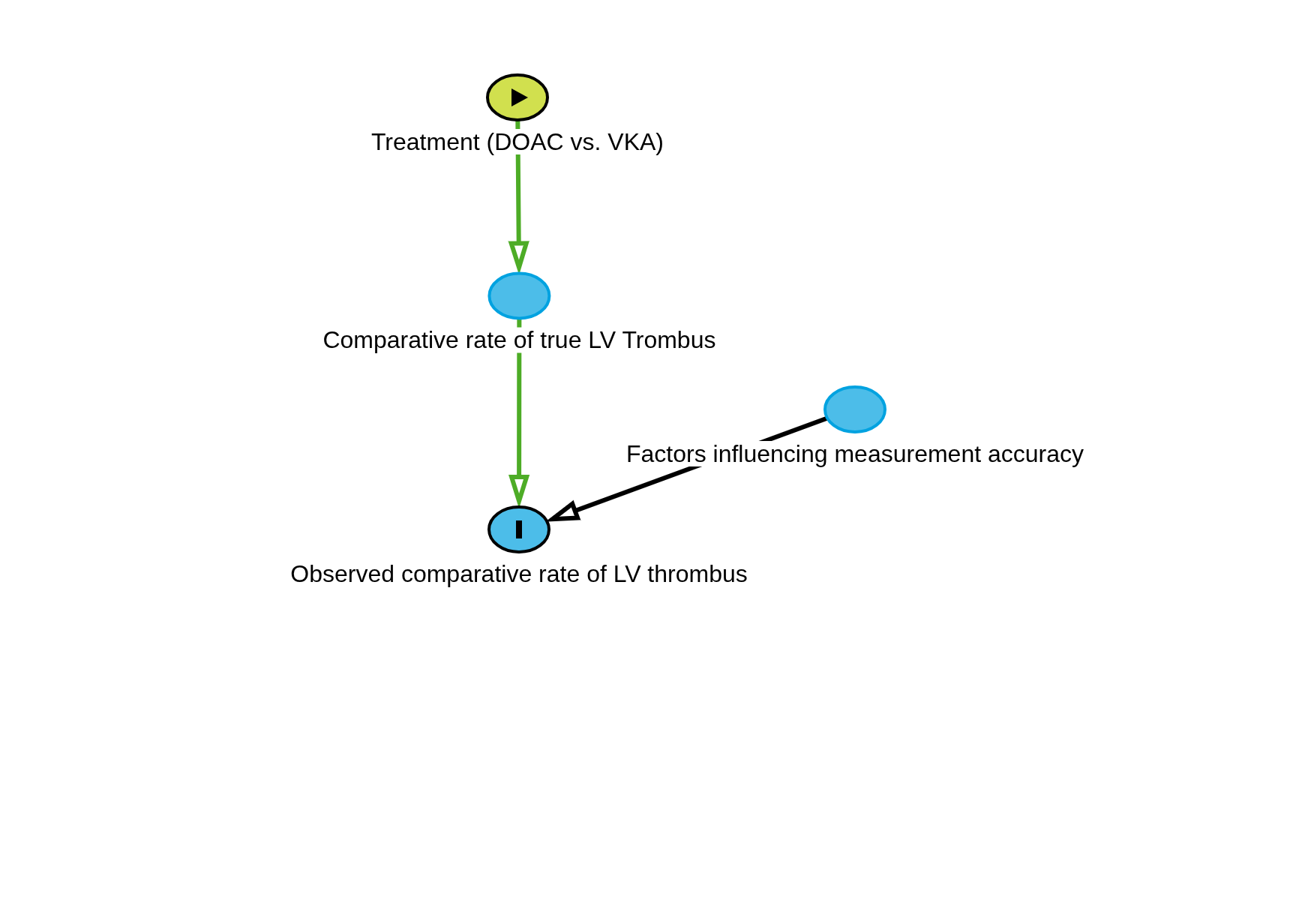
**

**Figure Legend:** The causal diagram in Figure depicts the relations among treatment assignment (A: DOAC vs VKA), the true presence of LV thrombus (Y*), and the measured outcome based on non-contrast transthoracic echocardiography (Y). The diagram also includes measurement context variables (M), such as image quality, use of contrast, and timing of imaging. Under this diagram, the causal effect of interest is that of A on Y*. However, because Y* is not directly observed, analyses use the measured outcome Y instead. As shown in the diagram, Y is a child of Y*, and its value is affected by M, which may itself be influenced by A. Consequently, the measured outcome Y is a misclassified proxy for the true outcome Y*. If M is independent of A given Y*—that is, if the use of contrast or image quality does not systematically differ by treatment group—then the misclassification of Y with respect to Y* is nondifferential. In this case, conditioning on Y instead of Y* leads to attenuation (bias toward the null) of the estimated effect of A on Y. This arises because measurement error in a binary outcome that is nondifferential with respect to exposure reduces the apparent difference in outcome probabilities between exposure groups. Thus, while the estimated effect of DOAC versus VKA on TTE-detected thrombus (Y) may approximate the causal effect on true thrombus (Y*) under nondifferential error, reliance on non-contrast imaging can introduce bias—typically toward the null.

**Supplementary Table 1 – Baseline Characteristics of Patients in Included Studies**

| **Study**    **Characteristics** | **Isa, W. Yus Haniff W. et al 2020**  **(N=27)** | | **Abdelnaby et al 2021 (No-LVT)**  **(N=79)** | | **Alcalai et al 2021**  **(N=35)** | | **Youssef et al 2021**  **(N=50)** | | **Mansouri et al 2024**  **(N=52)** | | **Jenab et al 2025**  **(REWARF-STEMI)**  **(N=50)** | | **Shah et al 2025**  **(RIVAWAR)** | |
| --- | --- | --- | --- | --- | --- | --- | --- | --- | --- | --- | --- | --- | --- | --- |
| **Imaging modality for diagnosis**  **and F/U of LVT** | **Non-contrast 2D TTE** | | **Non-contrast 2D TTE** | | **Non-contrast 2D TTE** | | **Non-contrast 2D TTE** | | **Non-contrast 2D TTE** | | **Non-contrast 2D TTE** | | **Non-contrast 2D TTE** | |
| **Follow-up time** | **3 months** | | **6 months** | | **3 months** | | **6 months** | | **3 months** | | **3 months** | | **3 months** | |
| **DOAC** | APIXABAN  (n=14) | WARFARIN (n=13) | RIVAROXABAN (n=39) | WARFARIN (n=40) | APIXABAN (n=18) | WARFARIN (n=17) | APIXABAN (n=25) | WARFARIN (n=25) | RIVAROXABAN (n=26) | WARFARIN (n=26) | RIVAROXABAN  (n =26) | WARFARIN (n=24) | RIVAROXABAN  (n =171) | WARFARIN  (n=90) |
| **Age* (years)** | 55.4 **±** 11 | 55 **±** 11.4 | 49.6 **±** 12.5 | 49.6 **±** 12.5 | 55.5 **±** 12.9 | 58.8 **±** 10.2 | 52 **±** 8.2 | 53 **±** 7.9 | 57.9 **±** 10.2 | 55.1 **±** 9.9 | 55 (50-60) | 55 (50-62.7) | 54.5 **±** 10.8 | 54.5 **±** 10.6 |
| **Male sex, n (%)** | 13 (93) | 12 (92) | 45 (57) | | 13 (72.2) | 15 (88.2) | NA | NA | 23 (88.5) | 21 (80.8) | 22 (84.7) | 19 (79.2) | 132 (77.2) | 75 (83.3) |
| **Smoking, n (%)** | NA | NA | NA | NA | 13 (72.2) | 10 (58.8) | 11 (44.0) | 12 (48.0) | 12 (46.1) | 11 (42.3) | 11 (42.3) | 10 (41.7) | 61 (35.7) | 44 (48.9) |
| **HTN, n (%)** | 8 (57.1) | 9 (69.2) | 42 (53.1) | | 7 (38.9) | 7 (41.2) | 11 (44.0) | 10 (40.0) | 10 (38.4) | 9 (34.6) | 9 (34.6) | 14 (58.3) | 111 (64.9) | 68 (75.6) |
| **DM, n (%)** | 7 (50.0) | 9 (69.2) | 42 (53.1) | | 8 (44.4) | 5 (29.4) | 12 (48.0) | 11 (44.0) | 11 (42.3) | 12 (46.1) | 7 (26.9) | 5 (20.8) | 73 (42.7) | 43 (47.8) |
| **Prior CKD, n (%)** | 5 (35.7) | 7 (53.8) | NA | NA | 3 (16.7) | 1 (5.9) | NA | NA | NA | NA | NA | NA | NA | NA |
| **Prior IHD, n (%)** | 9 (64.3) | 8 (61.5) | 31(78.5) | | 4 (22.2) | 3 (17.7) | 4 (16.0) | 5 (20.0) | NA | NA | 9 (34.6) | 6 (25.0) | NA | NA |
| **HAS-BLED score*** | 1.0 ± 0.68 | 1.46 ± 0.66 | NA | NA | NA | NA | 0.87 ± 0.82 | 0.85 ± 0.69 | NA | NA | NA | NA | NA | NA |
| **Baseline**  **EF* (%)** | 33.5 ± 5.73 | | 36.6 | | 35 ± 5 | 36 ± 7 | 26.4 ± 6.1 | 27.3 ± 9.2 | 29.6 ± 7.4 | 31.3 ± 8.3 | 32 (25-40) | 30 (25-35) | ≤35%^§^:  160 (93.6) | ≤35%^§^: 85 (94.4) |
| **Baseline thrombus size* (length, mm)** | NA | NA | 16.1 ± 4.4 | | 19.9 ± 9.4 | 18.5 ± 6.9 | 28.6 ± 9.6 | 25.6 ± 11.5 | NA | NA | 15 (9.75-18) | 18 (14-22.7) | NA | NA |
| **Baseline thrombus size *(width, mm)** | NA | NA | 11.5 ± 2.6 | | 12.4 ± 5.8 | 12.3 ± 4 | 15.4 ± 7.1 | 15.9 ± 6.4 | NA | NA | 8 (5-10) | 9 (5-17) | NA | NA |
| **Thrombus size (mm2)** | NA | NA | NA | NA | NA | NA | NA | NA | 190.3 ± 184.2 | 267.6 ± 223.9 | NA | NA | NA | NA |
| **Duration of triple therapy** | NA | | NA | | 30 days | | 90 days | | 30 days | | 07 days | | 30 days | |
| **Creatinine level*, mg/dL** | NA | NA | NA | NA | NA | NA | 1.2 ± 0.3 | 1.21 ± 0.51 | 1.2±0.2 | 1.2±0.3 | 1.1 (1.0-1.2) | 1.1 (0.9-1.3) | NA | NA |
| **Hemoglobin level*, gm/dL** | NA | NA | NA | NA | NA | NA | 13.5 ± 2.6 | 13.6 ± 2.7 | 13.1±2.3 | 13.4±2.5 | 14.8 (14.1-16.1) | 14.7 (13.2-15.7) | NA | NA |

**Legend:** Baseline characteristics of patients reported in each included study.

Abbreviations: BMI, body mass index; CKD, chronic kidney disease; DM, diabetes mellitus; EF, ejection fraction; F/U, follow-up; HTN, hypertension; IHD, ischemic heart disease; LVT, left ventricular thrombus; NA, not available; SD, standard deviation. ***** Data are presented as mean ± standard deviation (SD), median (interquartile range), or number (percentage), as applicable. ^§^ The RIVAWAR trial reports the number and percentage of participants with an EF ≤35% or >35%; here, we present the number and percentage of those with an EF ≤35%.

**Supplementary Table 2.** Sensitivity analyses of major outcomes using treatment-arm continuity correction, Hartung-Knapp-Sidik-Jonkman, risk difference, and generalized linear mixed model approaches.

| **Outcome** | **Method** | **Estimate** | **Lower CI** | **Upper CI** | ***p* value** | **I^2^** | **Tau^2^** | **Rare event** |
| --- | --- | --- | --- | --- | --- | --- | --- | --- |
| **ACM** | REML, CC=0.5 | 0.92 | 0.36 | 2.31 | 0.85 | 0 | 0 | No |
|  | TACC | 0.92 | 0.36 | 2.32 | 0.86 | 0 | 0 | No |
|  | HKSJ random-effects | 0.92 | 0.29 | 2.86 | 0.82 | 0 | 0 | No |
|  | Risk difference | 0.01 | -0.03 | 0.04 | 0.74 | 0 | 0 | Yes |
|  | GLMM (OR) | NA* | | | | | | Yes |
| **MACE** | REML, CC=0.5 | 0.5 | 0.16 | 1.54 | 0.23 | 0 | 0 | No |
|  | TACC | 0.5 | 0.16 | 1.55 | 0.23 | 0 | 0 | No |
|  | HKSJ random-effects | 0.5 | 0.09 | 2.94 | 0.3 | 0 | 0 | No |
|  | Risk difference | -0.02 | -0.08 | 0.03 | 0.39 | 0.29 | 0 | Yes |
|  | GLMM (OR) | NA* | | | | | | Yes |
| **Major bleeding** | REML, CC=0.5 | 0.54 | 0.2 | 1.48 | 0.23 | 0 | 0 | No |
|  | TACC | 0.54 | 0.2 | 1.48 | 0.23 | 0 | 0 | No |
|  | HKSJ random-effects | 0.54 | 0.17 | 1.74 | 0.22 | 0 | 0 | No |
|  | Risk difference | 0 | -0.03 | 0.03 | 0.82 | 0 | 0 | Yes |
|  | GLMM (OR) | 0.51 | 0.19 | 1.36 | 0.18 | NA | | Yes |
| **Rehospitalization** | REML, CC=0.5 | 1.36 | 0.47 | 3.94 | 0.58 | 0 | 0 | No |
|  | TACC | 1.36 | 0.47 | 3.94 | 0.58 | 0 | 0 | No |
|  | HKSJ random-effects | 1.36 | 0 | 909.6 | 0.66 | 0 | 0 | No |
|  | Risk difference | 0 | -0.07 | 0.06 | 0.93 | 0 | 0 | Yes |
|  | GLMM (OR) | NA* | | | | | | Yes |
| **Stroke/systemic embolism** | REML, CC=0.5 | 0.76 | 0.12 | 4.68 | 0.77 | 0.42 | 1.46 | No |
|  | TACC | 0.77 | 0.13 | 4.74 | 0.78 | 0.42 | 1.44 | No |
|  | HKSJ random-effects | 0.76 | 0.04 | 13.58 | 0.78 | 0.42 | 1.46 | No |
|  | Risk difference | 0 | -0.04 | 0.03 | 0.89 | 0.35 | 0 | Yes |
|  | GLMM (OR) | 0.36 | 0 | 26.73 | 0.64 | NA | | Yes |
| **Thrombus resolution** | REML, CC=0.5 | 1.02 | 0.95 | 1.09 | 0.59 | 0.09 | 0 | No |
|  | TACC | 1.02 | 0.95 | 1.09 | 0.59 | 0.09 | 0 | No |
|  | HKSJ random-effects | 1.02 | 0.94 | 1.11 | 0.62 | 0.09 | 0 | No |
|  | Risk difference | 0.04 | -0.03 | 0.11 | 0.27 | 0.26 | 0 | Yes |
|  | GLMM (OR) | 0.32 | 0.08 | 1.29 | 0.11 | NA | | Yes |

**Legends:** * The model did not converge due to sparse data and rare events.

**Abbreviations:** CC: continuity correction; CI: confidence interval; GLMM: Generalized Linear Mixed Model; HKSJ: Hartung-Knapp-Sidik-Jonkman; OR: odds ratio; REML: Restricted Maximum Likelihood; TACC: treatment-arm continuity correction.

**Supplementary Table 3.** Grading of Recommendations Assessment, Development, and Evaluation (GRADE) assessment.

| **Certainty assessment** | | | | | | | **Summary of findings** | | | | |
| --- | --- | --- | --- | --- | --- | --- | --- | --- | --- | --- | --- |
| **Participants (studies) Follow-up** | **Risk of bias** | **Inconsistency** | **Indirectness** | **Imprecision** | **Publication bias** | **Overall certainty of evidence** | **Study event rates (%)** | | **Relative effect (95% CI)** | **Anticipated absolute effects** | |
|  |  |  |  |  |  |  | **With Warfarin** | **With DOACs** |  | **Risk with Warfarin** | **Risk difference with DOACs** |
| **LV thrombus resolution at 3 months** | | | | | | | | | | | |
| 540 (7 RCTs) | Not serious | Not serious | Not serious | Serious^a^ | None | ⨁⨁⨁◯ Moderate^a^ | 181/228 (79.4%) | 271/312 (86.9%) | **RR 1.05** (0.95 to 1.16) | 181/228 (79.4%) | **40 more per 1,000** (from 40 fewer to 127 more) |
| **Major adverse cardiovascular events** | | | | | | | | | | | |
| 290 (6 RCTs) | Not serious | Not serious | Not serious | Serious^b^ | None | ⨁⨁⨁◯ Moderate^b^ | 11/143 (7.7%) | 4/147 (2.7%) | **RR 0.50** (0.16 to 1.54) | 11/143 (7.7%) | **38 fewer per 1,000** (from 65 fewer to 42 more) |
| **All-cause mortality** | | | | | | | | | | | |
| 472 (6 RCTs) | Not serious | Not serious | Not serious | Serious^b^ | None | ⨁⨁⨁◯ Moderate^b^ | 7/193 (3.6%) | 10/279 (3.6%) | **RR 0.92** (0.36 to 2.31) | 7/193 (3.6%) | **3 fewer per 1,000** (from 23 fewer to 48 more) |
| **Stroke or systemic emboli** | | | | | | | | | | | |
| 551 (7 RCTs) | Not serious | Not serious | Not serious | Serious^b^ | None | ⨁⨁⨁◯ Moderate^b^ | 8/233 (3.4%) | 7/318 (2.2%) | **RR 0.76** (0.12 to 4.76) | 8/233 (3.4%) | **8 fewer per 1,000** (from 30 fewer to 129 more) |
| **Major bleeding events** | | | | | | | | | | | |
| 541 (7 RCTs) | Not serious | Not serious | Not serious | Serious^b^ | None | ⨁⨁⨁◯ Moderate^b^ | 11/228 (4.8%) | 7/313 (2.2%) | **RR 0.54** (0.20 to 1.48) | 11/228 (4.8%) | **22 fewer per 1,000** (from 39 fewer to 23 more) |

#### **Explanations:**

#### a. Trial sequential analysis showed the cumulative Z-curve did not cross monitoring or futility boundaries and did not reach the required information size (RIS), indicating imprecision. The imprecision concern was also raised by the use of non-contrast transthoracic echocardiography (TTE) as the diagnostic modality to assess LV thrombus resolution in all included RCTs.

#### b. The confidence interval was wide, suggesting a possibility of no effect or a slight benefit or harm.

**Abbreviations:** CI: confidence interval; RR: risk ratio.
